# Supplementary material for: Heat adaptation of phage T7 under an extended genetic code
Source: Virus Evol. 2021 Dec 1;7(2):veab100. doi: 10.1093/ve/veab100 (PMC8923235; doi:10.1093/ve/veab100)
Supplement: veab100_Supp [file veab100_supp.zip › Sequencing_Analysis.zip!/Sequencing_Analysis/A1/evidence/RA_27.html]

BRESEQ :: Evidence


| Read alignment evidence... | | | | | | | | | | | |
| --- | --- | --- | --- | --- | --- | --- | --- | --- | --- | --- | --- |
|  | seq id | position | | ref | new | freq | score (cons/poly) | reads | annotation | genes | product |
| \* | NC\_001604.1 | 24,386 | 0 | G | T | 21.1% | 5664.8 / inf | 2802 | intergenic (–/–) | *–/–* | –/– |
| Reads supporting (aligned to +/- strand):  **ref** base G (1066/1123);  **new** base T (272/312);  **total** (1362/1440) | | | | | | | | | | | |
| Fisher's exact test for biased strand distribution *p*-value = 3.76e-01 | | | | | | | | | | | |
| Kolmogorov-Smirnov test that lower quality scores support variant *p*-value = 2.15e-01 | | | | | | | | | | | |

Only 100 of 2837 total aligned reads displayed.  

`AGGAGGCCGCACGCACCCGCGCTGCTAACAAAGCCCGAAAGGAAGCTGAGTTGGCTGCTGCCACCGCTGAGCAATAACTAGCATAACCCCTTGGGGCCTCTAAACGGGTCTTGAGGGGTTTTTTGCTGAAAGGAGGAACTATATGCGCTCATACGATATGAACGTTGAGACTGCCGCTGAGTTATCAGCTGTGAACGACATTCTGGCGTCTATCGGTGAACCTCCGGTATCAACGCTGGAAGGTGACGCTAACGCAGATGCAGCGAACGCTCGGCGTATTCTCAACAAGATTAACCGACAGATTCAATCTCGTGGATGGACGTTCAACATTGAGGAAGGCATAACGCTACTACCTGATGTTTACTCCAACCTGATTGTATACAGTGACGACTATTTATCCCTAATGTCTACTTCCGGTCAATCCATCTACGTTAACCGAGGTGGCTATGTGTATGACCGAACGAGTCAATCAGACCGCTTTGACTCTGGTATTACTGTGAACATTATTCGTCTCCGCGACTACGATGAGATGCCTGAGTGCTTCCGTTACTGGATTGTCACCAAGGCTTCCCGTCAGTTCAACAACCGATTCTTTGGGGCA  >  NC_001604.1/24086‑24686`  

                                                                                                                                                                                                                                                                                                            `|`                                                                                                                                                                                                                                                                                                              
   `aggCCGCACGCACCCGCGCTGCTAACAAAGCCCGAAAGGAAGCTGAGTTGGCTGCTGCCACCGCTGAGCAATAACTAGCATAACCCCTTGGGGCCTCTAAACGGGTCTTGAGGGGTTTTTTGCTGAATGGAGGAACTATATGCGCTCATGCGATATTAACGTTGAGACTGCCGCTGAGTTATCAGCTGTGAACGACATTCTTGCGTCTATCGGTGAACCTCCGGTATCAACGCTGGAAGGTGACGCTAACGCAGATGCAGCTAACGCGCGGCGTATTCTCACCAAGATTAACCGACATAtt                                                                                                                                                                                                                                                                                                           >  1:103923/1‑301 (MQ=255)`  
   `aggCCGCACGCACCCGCGCTGCTAACAAAGCCCGAAAGGAAGCTGAGTTGGCTGCTGCCACCGCTGAGCAATAACTAGCATAACCCCTTGGGGCCTCTAAACGGGTCTTGAGGGGAATTTTGCTGAAAGGAGGAACTATATGCGCCCATACGATATGAACGTTGAAACTGCCGCTGAGTTATCAGCTGTGAACGACCTTCTGGCGTCTATCGGTGAACCTCCGGTATCAACGCTGGAAGGTGACGCTAACGCAGAAGTAGCGAACGCTCGGCGTATTCTCCACAAGTTAAACCGACAGAtt                                                                                                                                                                                                                                                                                                           >  1:45405/1‑301 (MQ=255)`  
     `gCCGCACGCACCCGCGCTGCTAACAAAGCCCGAAAGGAAGCTGAGTTGGCTGCTGCCACCGCTGAGCAATAACTAGCATAACCCCTTGGGGCCTCTAAACGGGTCTTGAGGGGTTTTTTGCTGAAAGGAGGAACTATATGCGCTCATGCGATATGACCGTTGAGACTGCCGCTGCGTTATCAGCTGTTAACGACATTCTGGCGTCTATCGGTGAACCTCCGGTATCAACGCTGGAAGGTGACGCTAACGCAGATGCAGCGAACGCTAGGCGTATTCTCAACAAGATTAACCGACATATTCa                                                                                                                                                                                                                                                                                                         >  1:146023/1‑301 (MQ=255)`  
        `gcacgcacCCGCGCTGCTAACAAAGCCCGAAAGGAAGCTGAGTTGGCTGCTGCCACCGCTGAGCAATAACTAGCATAACCCCTTTGGGCCTCTAAACGGGTCTTGAGGGGTTTTTTGCTGAAACGAGGAACTATATGCGATCATACGATACTACCGTTGAGACTGCCGCTGAGTTATCAGCTGTGAACCACATTCTGGCGACTCTCGGTGAACCTCCGGTATCAACGCTGGAAGGTGACGCTAACGCAGATGTAGCGAACGCTCGGCGTAAGCTCAACAAGATTACCCGACAGATTCAAtc                                                                                                                                                                                                                                                                                                      >  1:354063/1‑301 (MQ=255)`  
         `cacgcacCCGCGCTGCTAACAAAGCCCGAAAGGAAGCTGAGTTGGCTGCTGCCACCGCTGAGCAATAACTAGCATAACCCCTTGGGGCCTCTAAACGGGTCTTGAGGGGTTTTTTGCTGAAAGGAGGAACTATATGCGCTCATACGATATGAACGTTGAGACTGCCGCTGAGTTATCAGCTGTGAACGACATTCTGGCGTCTATCGGTGAACCTCCGGTATCAACGCTGGAAGGTGACGCTAACGCAGATGTAGCGAACGCTCGGCGTATTCTCAACAAGATTAACCGACAGATTCAAtct                                                                                                                                                                                                                                                                                                     <  1:222001/301‑1 (MQ=255)`  
         `cacgcacCCGCGCTGCTAACAAAGCCCGAAAGGAAGCTGAGTTGGCTGCTGCCACCGATGAGCAATAACTAGCATAACCCCTTGGGGCCTCTAAACGGGTCTTGAGGGGTTTTTTGCTGAAAGGAGGAACTATATGCGCTCATGCGATATGAACGTTGAGACTGCCGCTGAGTTATCAGCTATGAACGACATTCTGGCGTCTATCGGTGAACCTCCGGTATCAACGCTGGAAGGTGACGCTAACGCAGATGCAGCGAACGCTCGGCGTATTCTCAACAAGATTAACCGACATATTCAAtct                                                                                                                                                                                                                                                                                                     <  1:131117/301‑1 (MQ=255)`  
            `gcacCCGCGCTGCTAACAAAGCCCGAAAGGAAGCTGAGTTGGCTGCTGCCACCGCTGAGCAATAACTAGCATAACCCCTTGGGGCCTCTAAACGGGTCTTGAGGGGTTTTTTGCTGAAAGGAGGAACTATATGCGCTCATACGATATGAACGTTGAGACTGCCGCTGAGTTATCAGCTGTGAACGACATTCTGGCGTCTATCGGTGAACCTCCGGTATCAACGCTGGAAGGTGACGCTAACTCAGATGTAGCGAACGCTCGGCGTATTCTCAACAAGATTAACCGACAGATTCAATCTCGt                                                                                                                                                                                                                                                                                                  <  1:462533/301‑1 (MQ=255)`  
             `cacCCGCGCTGCTCACAAAGCCCGAAAGTAAGCTGAGTTGGCTGCTGCCACCGCTGAGCAATCACTAGCATAACCCCTTGGGGCCTCTAAACGGGTCTTGAGGGGTTTTTTGCTGAAAGGAGGAACTATATGCGTTCATACGATATGAACGTTGAGACTGCCGCTGAGTTATCATCTGTGCACTCCTTTCTGGTGTCTTTCGTTGAACCTCCGGTATCAACGCTTCATGTTGATGCTAACTCAGATTTATCGTACGTTCTGCGTATCTCCAACCAACTTAACCAACTAATTCATtctcttt                                                                                                                                                                                                                                                                                                 >  1:265626/1‑298 (MQ=255)`  
                     `ctgctAACAAAGCCCGAAAGGAAGCTGAGTTGGCTGCTGCCACCGCTGAGCAATAACTAGCATAACCCCTTGGGGCCTCTAAACGGGTCTTGAGGGGTTTTTTGCTAAAAGGAGGAACTATATGCGCTCATACGATATGAACGTTGAGACTGCCGCTGAGTTATCAGCTGTGAACGACATTCTGGCGTCTATCGCTGAACCTCCGGTATCAACGCTGGAAGGTGACGCTAACGCAGATGTAGCGAACGCTCGGCGTATTCTCAACAAGATTAACCGACAGATTCAAtctctgcttcttata                                                                                                                                                                                                                                                                                         >  1:108355/1‑290 (MQ=255)`  
                       `gctAACAAAGCCCGAAAGGAAGCTGAGTTGGCTGCTGCCACCGCTGAGCAATAACTAGCATAACCCCTTGGGGCCTCTAAACGGGTCTTGAGGGGTTTTTTGCTGAAAGGAGGAACTATATGCGCTCATACGATATGAACGTTGAGACTGCCGCTGAGTTATCATCTGTGAACGACATTCTGGCGGCTATCGGTGAACCTCCGGTATCAACGCTGGAAGGTGACGCTAACGCAGATGTAGCGAACGCCCGGCCTCGTCTCAACAAGATAAACCGCCAGATTCCATCCCGtggacgaaccgt                                                                                                                                                                                                                                                                                       >  1:192783/1‑293 (MQ=255)`  
                            `cAAAGCCCGAACGGAATCTGATTTGGCTGCTGCCACCGCTGAGCAATAACTAGCATAGCCCCTTTGGGCCTCTCAACGGGTCTTGAGGGGTTTTTTGCTGAAAGTAGGAACTATATGCGCTCATACGATATGAACGTTGAGACTGCCGCTGAGTTATCAGCTGTTAACGACATTCTGGCGTCTATCGTTGATCCTCCGGTATCAACGCTGGAAGGTGACGCTAACGCAGCTGCAGCGAACGCTCGGCGTATTCTCAACAAGATTAACCGACATATTCAATCTCGTGGATGGACGTTCAACa                                                                                                                                                                                                                                                                                  <  1:213550/301‑1 (MQ=255)`  
                            `aaaaGCCCGAAAGGAAGCTGAGTTGGCTGCTGCCACCGCTGAGCAATAACTAGCATAACCCATTGGGGCCGCTAAACGGGTCTTGAGGGGTTTTTTGCTGAAAGGAGGAACTATATGCGCTCATACGATATGAACGTGGAGACTGCCGCTGAGTTATCAGCTGTGAACGACATTCTGGCGTCTATCGGTGAACCTCCGGTATCAACGCTGGAAGGTGACGCTAACGCAGATGTAGCGAACGCTCGGCGTATTCTCAACAAGATTAACCGACAGATTCAATCTCGTGGATGGACGTTCAACa                                                                                                                                                                                                                                                                                  <  1:134096/300‑1 (MQ=255)`  
                                  `ccGAAAGGAAGTTGAGTTGGCTGCTGCCACCGCTGAGCAATAACTAGCATAACCCCTTGGGGCCTCTAAACGGGTCTTGAGGGGTTTTTTGCTGAAAGGAGGAACTATATGCGCTCATACGATATGAACGTTGAGACTGCCGCTGAGTTATCAGCTGTGAACGACATTCTGGCGTCTATCGGTGAACCTCCGGTATCAACGCTGGAAGGTGACGCTAACGCAGATGTAGCGAACGCTCGGCGTATTCTCAACAAGATTAACCGACAGATTCAATCTCGTGGATGGACGTTCAACATTGAgg                                                                                                                                                                                                                                                                            <  1:73087/301‑1 (MQ=255)`  
                                  `ccGAAAGGAAGCTGAGTTGGCTGCTGCCACCGCTGAGCAATAACTAGCATAACCCCTTGGGGCCTCTAAACGGGTCTTGAGGGGTTTTTTGCTGAAAGGAGGAACTATATGCGCTCATACGATATGAACGTTGAGACTGCCGCTGAGTTATCAGCTGTGAACGACATTCTGGCGTCTATCGGTGAACCTCTGGTATCAACGCTGGAAGGTGACGCTAACGCAGATGTAGCGAACGCTCGGCGTATTCTCAACAAGATTAACCGACAGATTCAATCTCGTGGATGGACGTTCAACATTGAgg                                                                                                                                                                                                                                                                            <  1:178719/301‑1 (MQ=255)`  
                                  `ccGAAAGGAAGCTGAGTTGGCTGCTGCCACCGCTGAGCAATAACTAGCATAACCCCTTGGGGCCTCTAAACGGGTCTTGAGGGGTTTTTTGCTGAAAGGAGGAACTATATGCGCTCATACGATATGAACGTTGAGACTGCCGCTGAGTTATCAGCTGTGAACGACATTCTGGCGTCTATCGGTGAACCTCCGGTATCAACGCTGGAAGGTGACGCTAACGAAGATGTAGCGAACGCTCGGAGTATTCTCAACAAGATTAACCGACAGATTCAATCTCGTGGATGGACGTTCAACAtctgtc                                                                                                                                                                                                                                                                            >  1:269562/1‑296 (MQ=255)`  
                                        `ggAAGCTGAGTTGGCTGCTTCCACCGCTGAGCAATAACTAGCATAACCCCTTGGGGCCTCTAAACGGGTCTTGAGGGGTTTTTTGCTGAAAGGAGGAACTATATGCTCTCATACGATATGAACGTTGAGACTGCCGCTGAGTTATCAGATGTGAACGACTTTCTGGCGTCTATCGGTGAACCTCCGGTATCAACGCTGGAAGGTGACCCTAACGCAGCTGTATCGAACGCTCGGCGTATTCTCAACAACATTAACCGACAGATTCAATCTCTTGGATGGACGTTCAACATTGAGGAAGGCa                                                                                                                                                                                                                                                                      >  1:349174/1‑301 (MQ=255)`  
                                         `gaagcgtaagtGGCTGCTGCCAACGCTGGGCAAGAACTAGCATAGCCCCTTGGGGCCTTGTAACGGGTCTTGAGGGGTTTTTTGCTGAAAGGAGGAACTATATGCGCTCATACGATATGAACGTTGAGACTGCCGCTGAGTTATCAGCTGTGAACGAAATTCTAGCGTCTATCGTTGAACCTCCGGTATCAACGCTGGAAGGTGACGCTAACGCAGATGTAGCGAACGCTCGGCGTATTCTCAACAAGATTAACCGACAGATTAAATCTCGTGGATGGACGTTCAACATTGAGGAAGGCAt                                                                                                                                                                                                                                                                     <  1:48534/291‑1 (MQ=255)`  
                                             `cTGAGTTGGCTGCTGCCACCGCTGAGCAATAACTAGCATAACCCCTTGGGGCCTCTAAACGGGTCTTGAGGGGTTTTTTGCTGAAAGGAGGAACTATATGCGCTCATACGATATGAACGTTGAGACTGCCGCTGAGTTATCAGCTGTGAACGACATTCTGGCGTCTATCGGTGAACCTCCGGTATCAACGCTGGAAGGTGACGCTAACGCAGATACAGCGAACGCTCGGCGTATTCTCAACAAGATTAACCGACAGATTCAATCTCGTGGATGGACGTTCAACATTGAGGAAGGCATAACg                                                                                                                                                                                                                                                                 <  1:23835/301‑1 (MQ=255)`  
                                              `tGAGTTGGCTGCTGCCACCGCTGAGCAATAACTAGCATAACCCCTTGGGGCCTCTAAACGGGTCTTGAGAGGTTTTTTGCTGAAAGGAGGAACTATATGCGTTCATACGATATTAACGTTGAGACTGCCGCTGAGTTATCAGCTGTGAACGACATTCTGGCGTCTATCGGTGAACCTCCGGTATCAACGCTGGAAGGTGACGCTAACGCAGATGCAGCGAACGCTCGGCGTATTCTCAACAAGATTAACCGACAGATTCAATCTCGTGGATGGACGTTCAACATTGAGGAAGGCATAACGc                                                                                                                                                                                                                                                                <  1:18876/301‑1 (MQ=255)`  
                                              `ataagagacagctgcCACCGCTGAGCAATAACTAGCATAACCCCTTGGGGCCTCTAAACGGGTCTTGAGGGGTTTTTTGCTGAAAGGAGGAACTATATGCGCTCATACGATATGAACGTTGAGACTGCCGCTGAGTTATCAGCTGTGAACGACATTCTGGCGTCTATCGGTGAACCTCCGGTATCAACGCTGGAAGGTGACGCTAACGCAGATGTAGCGAACACTCGGCGTATTCTCAACAAGATTAACCGACAGATTCAATCTCGTGGATGGACGTTCAACATTGAGGAAGGCATAACGc                                                                                                                                                                                                                                                                <  1:435285/291‑1 (MQ=255)`  
                                                `aGTTGGTTGCTGCAGCCGCTGAGTAATAACGAGAATACCCCCTGGGGGCCTCTAAACTGAGCTTGA‑GGGTTTTTTGATGAAAGGAGGAACTAGATGCGCTCATGCGATATGAACGTTGAGACTGACGCTGAGTTATCAGCTGTGAACGACATTCTGGCGTCTATCGGTGAAACTCCGGTATCAACGCTGGAAGGTGACGCTAACGCAGATGCAGCGAACGCTCGGCGTATTCTCAACAAGATTAACCGACATATTCAATCTCGTGGATGGACGTTCAACATTGAGGAAGGAATAACGctac                                                                                                                                                                                                                                                             <  1:283881/301‑1 (MQ=255)`  
                                                        `gctgcCACCGCTGAGCAATAACTAGCATAACCCCTTGGGGCCTCTAAACGGGTCTTGAGGGGTTTTTTGCTGAAAGGAGGAACTATATGCGCTCATACGATATGAACGTTGAGACTGCCGCTGCGTTATCAGCTGTGAACGACATTATGGCGTCTATCGGTGAACCTCCGGTATCAACGCCGGAAGGCGACGCAAACGAAGATGTAGCGAACTCTCGGCGTATTCTCACCAAGATTAACCGAAAGATTCAATCCCGTGTAGGGACGTTCAACATTGTGGAAGGCAtcaaccaacacactgt                                                                                                                                                                                                                                                      >  1:367679/1‑286 (MQ=255)`  
                                                           `gaggcgcggagatgtgtataagagacagcccTTGGGGCCTCTAAACGGGGCTTGAGGGGTTTTTTGCTGAAAGGAGGAACTATATGCGCTCATACGATATGAACGTTGAGACTGCCGCTGAGTTATCAGCTGTGAACGACATTCTGGCGTCTATCGGTGAACCTCCGGTATCAACGCTGGAAGGTGACGCTAACGCAGATGTAGCGAACGCTCGGCGTATTCTCAACAAGATTAACCGACAGATTCAATCTCGTGGATGGACGTTCAACATTGAGGAAGGCATAACGCTACTACCTGATGt                                                                                                                                                                                                                                                   <  1:440423/273‑1 (MQ=255)`  
                                                               `ccGCTGAGCAATAACTAGCATAACCCCTTGGGGCCTCTAAACGGGTCTTGAGGGGTTTTTTGCTGAAAGGAGGAACTATATGCGCTCATACGATATGAACGTTGAGACTGCCGCTGAGTTATCAGCTGTGAACGACATTCTGGCGTCTATCGGTAAACCTCCGGTATCAACGCTGGAAGGTGACGCTAACGCAGATGTAGCGAACGATCGTCGTATTCTCAACAACATTAACCGCCAGATTCAATCCCGCGGACAGACGTTCAACATTGAGGAAGGCATAACGctactaacctgtctctta                                                                                                                                                                                                                                               >  1:322819/1‑289 (MQ=255)`  
                                                                    `gAGCAATAACTAGCATAACCCCTTGGGGCCTCTAAACGGGTCTTGAGGGGTTTTTTGCTGAAAGGAGGAACTATATGCGCTCATGCGATATGAACGTTGAGACTGCCGCTGAGTTATCAGCTGTGAACGACATTCTGGCGTCTATCGGTGAACCTCCGGTATCAACGCTGGAAGGTGACGCTAACGCAGATGCAGCGAACGCTCGGCGTATTCTCAACAAGATTAACCGACAGATTCAATCTCGTGGATGGACGTTCAACCTTGAGGAAGGCATAACGCTACTACCTGATGTTTACTcctg                                                                                                                                                                                                                                          >  1:169465/1‑299 (MQ=255)`  
                                                                    `gAGCAATAACTAGCATAACCCCTTGGGGCCTCTAAACGGGTCTTGAGGGGTTTTTTGCTGAAAGGAGGAACTATATGCGCTCATACGATATGAACGTTGAGACTGCCGCTGAGTTATCAGCTGTGAACGACATTCTGGCGTCTATCGGTGAACCTCCGGTATCAACGCTGGAAGGTGACGCTAACGCAGATGTAGCGAACGCTCGGCGTATTCTCAACAAGATTAACCGACAGATTCAAACTCGTGGATGGACGTTCAACATTGAGGAAGGCATAACGCTACTACCTgtctcttatacaca                                                                                                                                                                                                                                          >  1:184730/1‑288 (MQ=255)`  
                                                                    `gAGCAATAACTAGCATAACCCCTGGGGGCCTCTAAACGGGTCTTTAGGGGTTTTTTGCTGAAAGGAGGAACTATATGCGCTCATCCGCTATGCCCGTTGAGACTGCCGCTGAGTGATCAGCTGTGAACGACATTCTGGCGTCTATCGGTGAACCTCCGGCATCCACGCTAGAAGCTGCCGCTAACGACGTTGTAGCGCACTAACGGTGTATTCTCTACAAGCTTAACCGACAGATTTAATCTCGGTGATTGAAGTTCAACATTTCCGCAGTCATAACGAAACAACCTGATTTTCACTccca                                                                                                                                                                                                                                          >  1:161668/1‑299 (MQ=255)`  
                                                                     `ggtaatactgtgaacattattcgtctgCCTCTAAACGGGTCTTGAGGGGTTTTTTGCTGAAAGGAGGAACTATATGTGCTCATACGATATGAACGTTGAGACTGCCGCTGAGTTATCAGCTGTGAACGACATTCTGGCGTCTATCGGTGAACCTCCGGTATCAACGCTGGAAGGTGACGCTAACGCAGATGTAGCGAACGCTCGGCGTATTCTCAACAAGATTAACCGACAGATTCAATCTCGTGGATGGACGTTCAACATTGAGGACGGCATAACGCTCCTACCTGATGTTTACTCCAAc                                                                                                                                                                                                                                         >  1:199904/27‑301 (MQ=255)`  
                                                                          `tAACTAGCATAACCCCTTGGGGCCTCTAAACGGGTCTTGAGGGGTTTTTTGCTGAAAGGAGGAACTATATGCGCTCATGCGATATGAACGTTGAGACTGCCGCTGAGTTATCAGCTGTGAACGACATTCTGGCGTCTATCGGTGAACCTCCGGTATCAACGCTGGAAGGTGACGCTAACGCAGATGCCGCGGACGCTCGGCGTATTCTCAACAAGATTAACCGACATATTCAATCTCGTGGATGGACGTTCTACATTGAGGAAGGCATAACGCTACTACCTGATGTTTACTCCAACCTGAt                                                                                                                                                                                                                                    >  1:205941/1‑301 (MQ=255)`  
                                                                          `tAACTAGCATAACCCCTTGGGGCCTCTAAACGGGTCTTGAGGGGTTTTTTGCTGAAAGGAGGAACTATATGCGCTCATACGATATGAACGTTGAGACTGCCGCTGAGTTATCAGCTGTGAACGACATTCTGGCGTCTATCGGTGAACCTCCGGTATCAACGCTGGAAGGTGACGCTAACTCAGATGTAGCGAACGCTCGGCGTATTCTCAACAAGATTAACCGACAGATTCAATCTCGTGGATGGACGTTCAACATTGAGGAAGGCATAACGCTACTACCTGCTGTTTACTCCAACCTGAt                                                                                                                                                                                                                                    >  1:395086/1‑301 (MQ=255)`  
                                                                                `gCATAACCCCTTGGGGCCTCTAAACGGGTCTTGAGGGGTTTTTTGCTGAAAGGAGGAACTATATGCGCTCATACGATATGAACGTTGAGACTGCCGCTGAGTTATCAGCTGTGAACGACATTCTGGCGTCTATCGGTGAACCTCCGGTATCAACGCTGAAAGGTGACGCTAACGCAGATGTAGCGAACGCTAGGCGTATTCTCAACAAGATTAAACGACAGATTCAATCTCGTGGATGGACGTTCACCATTGAGGAAGTCATAACGCTAACACCTGATGTTTACTCCAACCTGATTGtata                                                                                                                                                                                                                              >  1:416634/1‑301 (MQ=255)`  
                                                                                 `tgtttatatgagacagctctAATAGGGTCTTGAGGGGTTTTGTGCTGAAAGGAGGAACTATATTCGCTCATACGCTATGAACGTTGAGACTGCCGGTGAGTTATCAGCTTTGAACGACATTCTGGCGTCTATCGGTGAACCTCCGGTATCAACGCTGGAAGGTGACGCTAACTCAGATGTAGCGAACGCTCGGCGTATTCTCAACAAGATTAACCGACAGATTCAATCTCGTGGATGGACGTTCAACATTGAGGAAGGCATAACGCTACTACCTGATGTTTACTCCAACCTGATTGTATAc                                                                                                                                                                                                                             <  1:76730/285‑1 (MQ=255)`  
                                                                                 `tgggtataagagacagctctAAACTGTTCTTGAGTGGTTTTTTGCTTAAAGGTGGAACTATATGCGCTCATACGATATGAACGTTGAGACTGCCGCTGAGTTATCAGCTGTGAACGACATTCTTGCGTCTATCGGTGAACCTCCGGTATCAACGCTGGAAGGTGACGCTAACGCAGATGTAGCGAACGCTCGGCGTATTCTCAACAAGATTAACCGACAGATTCAATCTCGTGGATGGACGTTCAACATTGAGGAAGGCATAACGCTACTACCTGATGTTTACTCCAACCTGATTGTATAc                                                                                                                                                                                                                             <  1:56094/285‑1 (MQ=255)`  
                                                                                 `cctAACCCCTTGGGGCCTCTAAACGGGTCTTGAGGGGTTTTTTGCTGAAAGGAGGAACTATATGCGCTCATACGATATGAACGTTGAGACTGCCGCTGAGTTATCAGCTGTGAACGACATTCTGGCGTCTATCGGTGAACCTCCGGTATCAACGCTGGAAGGTGACGCTAACGCAGATGTAGCGAACGCTCGGCGTATTCTAAACAAGATTAACCGACAGATTCAATCTAGTGGATGGACGTTCAACATTGAGGAAGGCATAACGCTACTACCTGATGTTTACTCCAACCTGATTGTATAc                                                                                                                                                                                                                             <  1:385143/299‑1 (MQ=255)`  
                                                                                   `aacccccTTGGGGCCTCTAAACGGGTCTTGAGGGGTTTTTTGCTGAAAGGAGGAACTATATGCGCTCATACGATATGAACGTTGAGACTGCCGCTGAGTTATCAGCTGTGAACGACATTCTGGCGTCTATCGGTGAACCTCCGGTATCAACGCTGGAAGGTGACGCTAACGCAGATGTAGCGAACGCTCGGCGTATTCTCAACAAGATTAACCGACAGATTCAATCTCGTGGATGGACGTTCAACATTGAGGAAGGCATAACGCTACTACCTGATGTTTACTCCAACCTGATTGTATACAg                                                                                                                                                                                                                           <  1:480732/298‑1 (MQ=255)`  
                                                                                    `aataagagacagacgctAAAAGGGTCTTGAGGGGTTTTTTGCTGAAAGGAGGAACTATATGCGCTCATACGATATGAACGTTGAGACTGCCGCTGAGTTATCAGCTGTGAACTACATTCTGGCGTCTATCGGTGAACCTCCGGTATCAACGCTGGAAGGTGACGCTAACGCAGATGTAGCGAACGCTCGGCGTATTCTCAACAAGATTAACCGACAGATTCAATCTCGTGGATGGACGTTCAACATTGAGGAAGGCATAACGCTACTACCTGATGTTTACTCCAACCTGATTGTATACAGt                                                                                                                                                                                                                          <  1:412315/286‑1 (MQ=255)`  
                                                                                      `ccccTGTGGGCCTATAAACGGGTGTGGAGGGGTGTTTTGCTGAAAGGAGGAACTATATGCGCTAATACGATATGAACGTTGAGACTGCCGCTGAGTTATCAGCTGTGAACGACATTCTGGCGTCTATCGGTGAACCTCCGGTATCAACGCTGGAAGGTGACGCTAACGCAGATGTAGCGAACGCTCGGCGTATTATCAACAAGATTAACCGACAGATGCAATCTCGTGGATGGACGTTCAACATTGAGGAAGGCATAACGCTACTACCTGATGTTTACTCCAACCTGATTGTATACAGTga                                                                                                                                                                                                                        <  1:399559/301‑1 (MQ=255)`  
                                                                                          `ttGGGGCCTCGAAACGGGTCTTGAGGGGTTTTTTGCTGAAAGGAGGAATTATATGCGCTCATACGATATGAACGTTGAGACTTCCGCTGAGTTATCAGCTGTGAACGACATTCTGGCGTCTATCGGTGAACCTCCGGTATCAACGCTGGAAGGTGACGCTAACGCAGATGTAGCGAACGCTCGGCGTATTCTCAACAAGATTAACCGACAGATTCAATCTCGTGGATGGACGTTCAACATTGAGGAAGGCATAACGCTACTACCTGATGTTTACTCCAACCTGATTGTATACAGTgacgac                                                                                                                                                                                                                    <  1:138221/301‑1 (MQ=255)`  
                                                                                          `gtggatccTCGAAACTGGGCTTGAGGGGTTGTTTGCTGAAAGGAGGAACTATATGAGCGCATGCGATATGAACGTGGATACTGACGCTGAGTTATCAGGTGTGAACGACATTCTGGCGTCTATCTGTGAACCTCCGGTATAAACGCTGGAAGGTGACGATAACGCAGATGAAGCGAACGCTCGGCGTATTCTCAACAAGATTAACCGACAGATTCAATCTCGTGGAGGGACGTTCAACATTGAGGAAGGCATAACGCTACTACCTGATGTTTACTCCAACCTGATTGTATACAGTgacgac                                                                                                                                                                                                                    <  1:505268/295‑1 (MQ=255)`  
                                                                                           `tGGGGCATCTAAACGGGTCTTGAGGGGTTTTTTGCTGAAAGGAGGAACTATATGCGCTCATACGATATGAACGTTGAGACTGCCGCTGAGTTATCAGCTGTGAACGACATTCTGGCGTCTATCGGTGAACCTCCGGTATCAACGCTGGAAGGTGACGCTAACGCAGATGTAGCGAACGCTCGGCGTATTCTCAACAAGATTAACCGACAGATTCAATCTCGTGGATGGACGTTCAACATTGAGGAAGGCATAACGCTACTACCTGATGTTTACTCCAACCTGATTGTATACAGTGACGACt                                                                                                                                                                                                                   <  1:181984/301‑1 (MQ=255)`  
                                                                                               `gCCTCTAAACGGGTCTTGGAGGGTTTTTTGCTGAAAGGGGGAAATATTTGCGTTCATAAGATATGAACGTTGAGAATGCCGCTGAGTTATCAGCTGTGAACGACATGCTGGCGTCTATCGGTGAACCTCCGGTATCAACGCTGGAAGGTGACGCTAACGCAGATGTAGCGAACGCTCGGCGTATTCGCAACAAGATTAACCGACAGATGCAATCTTGTGGATGGACGTTAAACATTGAGGAAGGCATAACGATACTACCTGATGTGTACTACAACCTGATTGTATACAGTGACGACTAttt                                                                                                                                                                                                               <  1:289474/301‑1 (MQ=255)`  
                                                                                                `ccTCTAAACGGGTCTTGAGGGGTTTTTTGCTGAAAGGAGGAACTATATGCGCTCATACGATATGAACGTTGAGACTGCCGCTGAGTTATCAGCTGTGAACGACATTCTGGCGTCTATCGGTGAACCTCCGGTATCAACGCTGGAAGGTGACGCTAACGCAGATGCAGCGAACGCTCGGCGTATTCTCAACAAGATTAACCGACATATTCAATCTCGTGGATGGACGTTCAACATTGAGGAAGGCCTAACCCTACTACCTGATGTTTACTCCAACCTGATTGTATAcctctctcttatacaa                                                                                                                                                                                                              >  1:391555/1‑286 (MQ=255)`  
                                                                                                  `tccaaaCGGGTCTTGAGGGGTTTTTTGCTGGAAGGATGAACTATATGCGCTAAAACGATATGAACGTTGAGACTGCCGCTGAGTTATCAGCTGTGAACGACATTCTGGCGTCTATCGGTGAACCTCCGGTATCAACGCTGGAAGGTGACGCTAACGCAGATGTAGCGAACGATCGGCGTATTCTCAACAAGATTAACCGACAGATTCAATCTCGTGGATGGACGTTCAACATTGAGGAAGGCATAACGCTACTACCTGATGTTTACTCCAACCTGATTGTATACAGTGACGACTATTTATc                                                                                                                                                                                                            <  1:472182/298‑1 (MQ=255)`  
                                                                                                     `aaaCGGGTCTTGAGGGGTTTTTTGCTGAAAGGAGGAACTATATGCGCTCATACGATATGAACGTTGAGACTGCCGCTGAGTTATCAGCTGTGAACGACATTCTGGCGTCTATCGGTGAACCTCCGGTATCAACGCTGGAAGGTGACGCTAACTCAGATGTAGCGAACGCTCGGCGTATGCTCAACAAGATTAACCGACAGATTCAATCTCGTGGATGGACGTTCAACATTGAGGAAGGCATAACGCTACTACCTGATGTTTACTACAACCTGATTGTATACAGTCACGACTATTTATcccc                                                                                                                                                                                                         >  1:16439/1‑300 (MQ=255)`  
                                                                                                          `ggTCTTGAGGGGTTTTTTGCTGAAAGGAGGAACTATATGCGCTCATACGATATGAACGTTGAGGCTGCCGCTGAGTTATCAGCTGTGAACGACATTCTGGCGTCTATCGGTGAACCTCCGGTATCAACGCTAGAAGGTGACGCTAACGCAGATGTAGCGAACGCTCGGCGTATTCTCAACAAGATTATCCGACAGATTCAATCTCGTGGATGGACGTTCAACATTGAGGAAGGCATAACGCTACTACCTGATGTTTACTCCAACCTGATTGTATACAGTGACGACTATTTATCCCTAATGt                                                                                                                                                                                                    <  1:31506/301‑1 (MQ=255)`  
                                                                                                            `tCTTGAGGGGTTTTTTGCTGAAAGGAGGAACTATATGCGCTCATACGATATGAACGTTGAGACTGCCGCTGAGTTATCAGCTGTGAACGACATTCTGGAGTCTATCGGTGAACCTCCGGTATCAACGCTTGAAGGTGACGCTAACGCAGATGTAGCGAACGCTCGGCGTATTCTCAACAAGATTAACCGACATATTCAATCTCGTGGATGGACGTTCAACATTGAGGAAGGCATAACGCTACTACCTGATGTTTACTCCAACCTGATTGTATACAGTGACGACTATTTATCCCTAATGTCt                                                                                                                                                                                                  <  1:34431/301‑1 (MQ=255)`  
                                                                                                                     `tgtataagagacagggaggaACTATATGCGCTCATATGATATGAACGTTGAGACTGCCGCTGAGTTATCAGCTGTGAACGACATTCTGGCGTCTATCGGTGAACCTCCGGTATCAACGCTGGAAGGTGACGCTAACGCAGATGTAGCGAACGCTCGGCGTATTCTCAACAAGATTAACCGACAGATTCAATCTCGTGGATGGACGTTCAACATTGAGGAAGGCATAACGCTACTACCTGATGTTTACTCCAACCTGATTGTATACAGTGACGACTATTTATCCCTAATGTCTACTTCCGGt                                                                                                                                                                                         <  1:404906/287‑1 (MQ=255)`  
                                                                                                                          `ttGCTGAAAGGAGGAACTATATGCGCTCATACGATATGAACGTTGAGACTGCCGCTGAGTTATCAGCTGTGAACGACATTCTGGCGTCTATCGGTGAACCTCCGGTATCAACGCTGGAAGGTGACGCTAACGCAGATGTAGCGAACGCTCGGCGTATTCTCAACAAGATTAACCGACAGATTCAATCTCGTGGATGGACGTTCAACATTGAGGAAGGCATAACGCTACTACCTGATGTTTACTCCAACCTGATTGTCTACAGTGACGACTATTTATCCCTAATGTCTACTTCCGGTCAATc                                                                                                                                                                                    >  1:85023/1‑301 (MQ=255)`  
                                                                                                                             `aagacaggaggAACTATATGCGCTCATACGATATGAACGTTGAGACTGCCTGTGAGTTATCAGCTGTGAACGACATTCTGGCGTCTATCGGTGAACCTCCGGTATCAACGCTGGAAGGTGACGCTAACGCAGATGTAGCGAACGCTCGGCGTATTCTCAACAAGATTAACCGACAGATTCAATCTCGTGGAAGGACGTTCAACATTGAGGAAGGCATAACGCTACTACCTGATGTTTACTCCAACCTGATTGTATACAGTGACGACTATTTATCCCTAATGTCTACTTCCGGTCAATACAt                                                                                                                                                                                 <  1:339210/296‑1 (MQ=255)`  
                                                                                                                                `gggctcggagatgtgtataagagacagATATGACCGGTGAGACTGCCGCTGAGTTATCAGCTGTGAACGACATTCTGGCGTCTATCGGTGAACCTCCGGTATCAACGCTGGAATGTGACGCTAACGCAGATGTAGCGAACGCTCGGCGTATTCTCAACAAGATTAACCGACAGATTCAATCTCGTGGATGGACGTTCAACAATGAGGAAGGCATAACGCTACTACCTGATGTTTACTCCAACCTGATTGTATACAGTGACGACTATTTATCCCTAATGTCTACTTCCGGTCAATCCATCTa                                                                                                                                                                              <  1:381545/275‑1 (MQ=255)`  
                                                                                                                                 `aaGGAGGAACTATATGCGCTCATACGATATGAACGTTGAGACTGCCGCTGAGTTATCAGCTGTGAACGACATTCTGGCGTCTATCGGTGAACCTACGGTATCAACGCTGGAAGGTGACGCTAACGCAGATGTAGCGACCGCTCGGCGTATTCTCAACAAGATTAACCTACAGATTCAATCTCGTGGATGGACGTTCAACATTGAGGAAGGCATAACGCTACTACCTGATGTTTACTCCAACTTGATTGTATACAGTGACGACTATTTATCCCTAATGTCTACTTCCggcccggtctcttat                                                                                                                                                                             >  1:244274/1‑288 (MQ=255)`  
                                                                                                                                  `aggaggAACTATATGCGCTCATACGATATGAACGTTGAGACTGCCGCTGAGTTATCAGCTGTGAACGACATTCTGGCGTCTATCGGTGAACCTCCGGTATCAACGCTGGAAGGTGACGCTAACGCAGATGTAGCGCACGCTCGACGTATTCTCAACAAGATTCACCGACATATTCAATCTCGTGGATGGACGTTCAACATTGAGTAAGCCCTAACGCTACTATCACATGTTTACTCCCACCTGTTTGTATACAGTAACGACTATTTATCCATACTCTCTACTCCCGGTCAATCCACCTAcc                                                                                                                                                                            >  1:261407/1‑300 (MQ=255)`  
                                                                                                                                   `ggaggaACTATATGCTCTCATGCGATATGATCGTTGAGACTGCCGCTGAGTTATCAGCTGTGAACGACATTCTGGCTTCTATCGGTGAACCTCCGGTATCAACGCTGGAAGGTGACGCTAACGCATATGCAGCGAACGCTCGGCGTATTCTCAACAAGATTAACCGACATATTCAATCTCTTGGATGGACGTTCAACATTGAGTAAGGCATAACGCTACTACCTCATGTTTACTCCAACCTGATTGTATACAGTGACGACTATTTATCCCTAATGTCTACTTCCGGTCAATCCATCTACGt                                                                                                                                                                           <  1:67774/301‑1 (MQ=255)`  
                                                                                                                                   `acaggaACTATATGCGCTCATACGATATGAACGTTGAGACTGCCGCTGAGTTATCAGCTGTGAACGACATTCTGGCGTCTATCGGTGAACCTCCGGTATCAACGCTGGAAGGTGACGCTAACGCAGATGTAGCGAACGCTCGGCGTATTCTCAACAAGATTAACCGACAGATTCAATCTCGTGGATGGACGTTCAACATTGAGGAAGGCATAACGCTACTACCTGATGTTTACTCCAACCTGATTGTATACAGTGACGACTATTTATCCCTAATGTCTACTTCCGGTCAATCCATCTACGt                                                                                                                                                                           <  1:225773/299‑1 (MQ=255)`  
                                                                                                                                    `gacagaCTATATGCGCTCATGCGATATGAACGTTGAGACTGCCGCTGAGTTATCAGCTGTGAACGACATTCTGGCGTCTATCGGTGAACCTCCGGTATCAACGCTGGAAGGTGACGCTAACTCAGATGCAGCGAACGCTCGGCGTATTCTCAACAAGATTAACCGACATATTCAATCTCGTGGATGGACGTTCAACATTGAGGAAGGCATAACGCTACTACCTGATGTTTACTCCAACCTGATTGTATACAGTTACGACTATTTATCCCTAATGTCTACTTCCGGTCAATCCATCTACGtt                                                                                                                                                                          <  1:30064/296‑1 (MQ=255)`  
                                                                                                                                       `gaACTATATGCGCTCATACGATATGAACGTTGAGACTGCCGCTGAGTTATCAGCTGTGAACGACATTCTGGCGTCTATCGGTGAACCTACGGTATCAACGCTGGAAGGTGACGCTAACGCAGATGTAGCGAACGCTCGGCGTATTCTCAACAAGATTAACCGACAGATTCAATCTCGTGGATGGACGTTCAACATTGAGGAAGGCATAACGCTACTACCTGATGTTTACTCCAACCTGATTGTATACAGTGACGACTATTTATCCCTAATGTCTACTTCCGGTCAATCCATCTACGTTAAc                                                                                                                                                                       >  1:445579/1‑301 (MQ=255)`  
                                                                                                                                       `aaaagatatggtgtaaTACGATATGAACGTTGAGACTGCCGCTGAGTTATCAGCTGTGAACGATAATCTGGAGTCTATCGGTGAACCTCCGGTATCAACGCGGGAAGGTGACGCTAACGCAGATGTAGCGAACGCTCGGCGTATTCTCAACAAGATTAACCGACAGATTCAATCTCGTGGATGGACGTTCAACATTGAGGAAGGCATAACGCTACTACCTGATGTTTACTCCAACCTGATTGTATACAGTGACGACTATTTATCCCTAATGTATACTTCCGGTCAATCCATCTACGTTAAc                                                                                                                                                                       <  1:258390/286‑1 (MQ=255)`  
                                                                                                                                        `aaCTATATGCGCTCATGCGATGTGAACGTTGAGACTGCCGCTGAGTTATCAGCTGTGAACGACATTCTGGCGTCTATCGGTGAACCTCCGGTATCCACGCTGGAAGGTGACGCTAACGCAGATGCAGCGAACGCTCGGCGTATTCTCAACAAGATTAACCGACATATTCAATCTCGTGGCTGGACGTTCAACATTGAGGAAGGCATAAAGCTAGTACCTGATGTTTTCTCCAACCTGATTGTATACAGTGACGGCAATTTATCCTTAATGTCTACTTTCGGTCAATCCATCTACGTTAAcc                                                                                                                                                                      >  1:117182/1‑301 (MQ=255)`  
                                                                                                                                        `aaCTATATGCGCTCATGCGATATGAACGTTGAGACTGCCGCTGAGTTATCAGCTGTGAACGACATTCTGGCGTCTATCGGTGAACCTCCGGTATCAACGCTGGAAGGTGACGCTAACGCAGATGCAGCGAACGCTCGGCGTATTCTCAACAAGATTAACCGACATATTCAATCTCGTGGATGGACGTTCAACATTGAGGAAGGCATAACGCTACTACCTGATGTTTACTCCAACCTGATTGTATACAGTGACGACTATTTATCCCTAATGTCTACTTCCGGTCAATCCATCTACGTTAAcc                                                                                                                                                                      >  1:24759/1‑301 (MQ=255)`  
                                                                                                                                                 `cgcTCATACGATATTAACGTTGAGACTGCCGCTGAGTTATCAGCTGTGAACGACATTCTGGCGTCTATCGGTGAACCTCCGGTATCAACGCTGGAAGGTGACGCTAACGCAGATGTAGCGAACGCTCGGCGTATTCTCAACAAGATTAACCGACAGATTCAATCTCGTGGATGGACGTTCAACATTGAGGAAGGCATAACGCTACTACCTGATGTTTACTCCAACCTGATTGTATACAGTGACGACTATTTATCCCTAATGTCTACTTACGGTCAATCCATCTACGTTAACCGAGGTGGCt                                                                                                                                                             >  1:273477/1‑301 (MQ=255)`  
                                                                                                                                                  `gcTCATACGATATGAACGTTGAGACTGCCGCTGAGTTATCAGCTGTGAACGACATTCTGGCGTCTAACGGTGAACCTCCGGTATCAACGCTGGAAGGTGACGCTAACGCAGATGTAGCGAACGCTCGGCGTATTCTCAACAAGATTAACCGACAGATTCAATCTCGTGGATGGACGTTCAACATTGAGGAAGGCATAACGCTACTACCTGATGTTTACTCCAACCTGATTGTATACAGTGACGACTATTTATCCCTAATGTCTACTTCCGGTCAATCCATCTACGTTAACCGAGGTGGCTa                                                                                                                                                            <  1:217925/301‑1 (MQ=255)`  
                                                                                                                                                   `cTCATACGATATGAACGTTGAGACTGCCGCTGAGTTATCAGCTGTGAACGACATTCTGGCGTCTATCGGTGAACCTCCGGTATCAACGCTGGAAGGTGACGCTAACGCAGATGTAGCGAACGCTCGGCGTCTTCTCAACAAGATTAACCGACAGATTCAATCTCGTGGATGGCCGTTCACCATTGAGGAAGGCATAACGCTACTACCTGATGTTTACTCCAACCTGATTGTATACAGTGACGACTATTTATCCCTAATTTCTACTTCCGGTAAATCCATCTCCGTTTACCGAGGTTGCTAt                                                                                                                                                           >  1:372034/1‑301 (MQ=255)`  
                                                                                                                                                    `tCATACGATATGAACGTGGAGACTACCGCGGAGTTATCAGATGTTAACGACATTCTGGCGTCTATCGGTGAACCTCCGGTATCAACGCTGGAAGGTGACGCTAACGCAGATGTAGCGAACGCTCGGCGTATTCTCAACAAGATTAACCGACAGATTCAATCTCGTGGATGGACGTTCAACATTGAGGAAGGCATAACGCTACTACCTGATGTTTACTCCAACCTGATTGTATACAGTGACGACTATTTATCCCTAATGTCTACTTCCGGTCAATCCATCTACGTTAACCGAGGTGGCTAtg                                                                                                                                                          <  1:278403/301‑1 (MQ=255)`  
                                                                                                                                                    `gcATGCGATATGAACGTTGAGACTGCCGCTGAGTGATCAGCTGGGAACGACATTCTGGCGTCTATCGGTGAACCTCCGGTATCAACGCTGGAAGGTGACGCTAACGCAGATGCAGCGAACGCTCGGCGTATTCTCAACAAGATTAACCGACATATTCAATCTCGTGGATGGACGTTCAACATTGAGGAAGGCATAACGCTACTACCTGATGTTTACTCCAACCTGATTGTATACAGTGACGACTATTTATCCCTAATGTCTACTTCCGGTCAATCCATCTACGTTAACCGAGGTGGCTAtg                                                                                                                                                          <  1:22866/300‑1 (MQ=255)`  
                                                                                                                                                      `aTACGATATGAACGTTGAGACTGCCGCTGAGTTATCAGCTGTGAACGACATTCTGGCGTCTATCGGTGAACCTCCGGTATCAACGCTGGAAGGTGACGCTAACGCAGATGTAGCGAACGCTCTGCGTATTCTCAACAAGATTAACCGACAGATTCAATCTCGTGGATGGACGTTCAACATTGAGGAAGGCATAACGCTACTACCTGATGTTTACTCCAACCTGATTGTATACAGTGACGACTATTTATCCCTAATGTCTACTTCCGGTCAATCCATCTACGTTAACCGAGGTGGCTAtgtg                                                                                                                                                        <  1:476608/301‑1 (MQ=255)`  
                                                                                                                                                       `tACGATATGAACGTTGAGACTGCCGCTGAGTTATCAGCTGTGAACGACATTCTGGCGTCTATCGGTGAACCTCCGGTATCAACGCTGGAAGGTGACGCTGACGCAGATGTAGCGAACGCTCGGCGTATTCTCAACAAGATTAACCGACAGATTCAATCTCGTGGCTGGACGTTCAACATTGAGGAAGGCATAACGCTACTACCTGATGTTTACTCCAACCTGATTGTATACAGTGACGACTATTTATCCCTACTGTCTACTTCCGGTCACTCCATCTACGTTAACCGAGGTGGCTAtgttt                                                                                                                                                       >  1:15567/1‑299 (MQ=255)`  
                                                                                                                                                         `cGATATTAACGTTGAGACTGCCGATGAGTTATCAGCTGTGAACGACATTCTGGCGTCTATCGGTGAACCTCCGGTATCAACGCTGGAAGGTGACGCTAACGCAGATGCAGCGAACGCTCGGCGTATTCTCAACAAGATTAACCGACATATTCAATCTCGTGGATGGACGTTCAACATTGAGGAAGGCATAACGCTACTACCTGATGTTTACTCCAACCTGATTGTATACAGTGACGACTATTTATCCCTAATGTCTACTTCCGGTAAATCCATCTACGTTAACCGAGGTGGCTAtgtgctg                                                                                                                                                     >  1:408603/1‑298 (MQ=255)`  
                                                                                                                                                          `gATATGAACGTTGAGACTGCCGCTGAGTTATCAGCTGTGAACGACATTCTGGCGTCTATCGGTGAACCTCCGGTATCAACGCTGGAAGGTGACGCTAACGCAGATGCAGCGAACGCTCGGCGTATTCTCAACAAGATTAACCGACATATTCAATCTCGTGGATGGACGTTCAACATTGAGGAAGGCATAACGCTACTACCTGATGTTTACTCCAACCTGATTGTATACAGTGACGACTATTTATCCCTAATGTCTACTTCCGGTCACTCCATCTACGTTAACCGAGGTGGcctgtctctta                                                                                                                                                    >  1:100304/1‑291 (MQ=255)`  
                                                                                                                                                           `atatGAACGTTGAGACTGCCGCTGAGTTATCATCTGTGAACGACATTCTGGCGTCTATCGGTGAACCTCCGGTATCAACGCTGGAAGGTGACGCTAACGCAGATGTAGCGAACGCTCGGCGTATTCTCAACAAGATTAACCGACAGATTCAATCTCGTGGATGGACGTTCAACATTGAGGAAGGCATAACGCTACTACCTGATGTTTACTCCAACCTGATTTTATACAGTGACGACTATTTATCCCTAATGTCTACTTCCGGTCAATCCATCTACGTTacacctgtctattataaacttct                                                                                                                                                   >  1:210330/1‑279 (MQ=255)`  
                                                                                                                                                           `atatGAACGTTGAGACTGCCGCTGAGTTATCAGCTGTGAACGACATTCTGGCGTCTATCGGTGAACCTCCGGTATCAACGCTGGAAGGTGACGCTAACGCAGATGTAGCGAACGCTCGGCGTATTCTCAACAAGATTAACCGACAGATTCAATCTCGTGGATGGACGTTCAACATTGAGGAAGGCATAACGCTACTACCTGATGTTTACTCCAACCTGATTGTATACAGTGACGACTATTTATCCCTAATGTCTACTTCCGGTCAATCCATCTACGTTAACCGAGGTGGCTATGTGTATGa                                                                                                                                                   >  1:421924/1‑301 (MQ=255)`  
                                                                                                                                                           `atatGAACGTTGAGACTGCCGCTGAGTTATCAGCTGTGAACGACATTCTGGCGTCTATCGGTGAACCCCCGGTATCAACGATGGAAGGTGACGCGAACGCAGATGTAGCGAACGCTCGGCGTATTATCAACAAGATTAACCGACAGAAGCAATCTCGGGGATGGACGTTCAACATTTAGGAATGCATAACGCTACTACCTGATGTTTACTCCAACCTGATTGTATACAGTGACGAATATTTATCCCTAATGTCAACTGCCGGACACTCCCTACACAGTAACAGAAGTAGCTAAGTGGATGa                                                                                                                                                   >  1:388173/1‑301 (MQ=255)`  
                                                                                                                                                            `tatGAACGTTGAGACTGCCGCTGAGTTATCAGCTGTGAACGACATTCTGGCGTCTATCGGTGAACCTCCGGTATCAACGCTGGAAGGTGACGCTAACGCAGATGTAGCGAACGCTCGGCGTATTCTCAACAAGATTAACCGACAGATTCAATCTCGTGGATGGACGTTCAACATTGAGGAAGGCATAACGCTACTACCTGATGTTTACTCCAACCTGATTGTATACAGTGACGACTATTTATCCCTAATGTCTACTTCCGGTCAATCCATCTACGTTAACCGAGGTGGCTATGTGTATGAc                                                                                                                                                  <  1:61196/301‑1 (MQ=255)`  
                                                                                                                                                            `aatGAGCGTGGCGACGGCCGCTGAGTTATCAGCTGTGAACGACTTTCTGGCTTCTATCGGTTAACCTCCAGTATCAACGCTGGAAGGTGACGCTAACGCAGATGTAGCGAACGCTCGGCGTATTCTTAACAAGATTAACCGGCAGATTCAAGCTCGTGGATGGACGTTCAACATTGAGGAAGGCATAACGCTACTACCTGATGTTTACTCCAACCTGATTGTATACAGTGACGACTATTTATCCCTAATGTCTACTTCCGGTCAATCCACCTAAGTTAACCGAGGTGGCTATGTGTATGAc                                                                                                                                                  <  1:252547/300‑1 (MQ=255)`  
                                                                                                                                                            `aatGAACGTTGAAACTGCCGCGTAGGTAGAAGCTGTGAACGACATTCTGGCGTCTATCGGTGAACCTCCGGTATCAACGCTGGAAGGCGACGCTAACGCAGATGTAGCGAACGCTCGGCGTAGTCTCAACAAGATTAACCGACAGATTCAATCTCGTGGATGGACGTTCAACATTGAGGAAGGCATAACGCTACTACCTGATGTTTACTCCAACCTGATTGTATACAGTGACGACTATTTATCCCTAATGTCTACTTCCGGTCAATCCATCTACGTTAACCGAGGTGGCTATGTGTATGAc                                                                                                                                                  <  1:89046/300‑1 (MQ=255)`  
                                                                                                                                                                          `tagCCTCTGATTTATCAGCTGTGAACGACATTCGGGCGTCTATCGGTGAACCTCCGGTATCAACGCTGGAAGGTGACGCTAACGCAGAAGTAGCGAACTCTCGTCGTATTCTCAACAAGATTAACCGACAGATTCAATCTCGTGGATGGACGTTCAACATCGAGGAAGGCATAACGCTACTACCTGTTGTTTACGCCAACCTGATTGTATACAGTGACGACTATTTATCCCTAATGTCTACTTCCGGTCAATCCATCTACGTTAACCGAGGTGGCTATGTGTATGACCGAACGAGTCAATc                                                                                                                                    <  1:80588/299‑1 (MQ=255)`  
                                                                                                                                                                                           `gCTGTTAACGACATTCTGGTGTCTATCGGTGAACCTCCGGTATCAACGCTGGAAGGTGACGCTAACGCAGATGTAGCGAACGCTCGGCGTATTCTCAACAAGATTAACCGACAGATTCAATCTCGTGGATGGACGTTCAACATTGAGGAAGGCATAACGCTACTACCTGATGTTTACTCCAACCTGATTGTATACAGTGACGACTATTTATCCCTAATGTCTACTTCCGGTCAATCCATCTACGTTAACCGAGGTGGCTATGTGTATGACAGAACGAGTCAATCAGACCGCTTTGACTCTg                                                                                                                   >  1:149608/1‑301 (MQ=255)`  
                                                                                                                                                                                           `gCTGTGAACGACATTCTGGCGTCTATCGGTGAACCTCCGGTATCAACGCTGGAAGGTGACGCTAACGCAGATGTAGCGAACGCTCGGCGTATTCTCGACAAGATTAACCGACAGATTCAATCTCGTGGATGGACGTTCAACATTGAGGAAGGCATAACGCTACTACCTGATGTTTACTCCAACCTGATTGTATACAGTGACGACTATTTATCCCTAATGTCTACTTCCGGTCAATCCATCTACGTTAACCGAGGTGGCTATGTGTATGACCGAACGAGTCAATCAGACCGCTTTGACTCTg                                                                                                                   >  1:9539/1‑301 (MQ=255)`  
                                                                                                                                                                                           `gCTGTGAACGACATTCTGGCGTCTATCGGTGAACCTCCGGTATCAACGCTGGAAGGTGACGCTAACGCAGATGTAGCGAACGCTCGGCGTATTCTCAACAAGATTAACCGACAGATTCAATCTCGTGGATGGACGTTCAACATTGAGGAAGGCATAACGCTACTACCTGATGTTTACTCCAACCTGATTGTATACAGTGACGACTATTTATCCCTAATGTCTACTTCCGGTCAATCCATCTACGTTAACCGAGGTGGCTATGTGTATGACCGAACGAGTCAATCAGACCGCTTTGACTCTg                                                                                                                   >  1:467605/1‑301 (MQ=255)`  
                                                                                                                                                                                           `gCTGTGAACGACATTCTGGCGTATATCGGTGAACCTCCGGTATCAACGCTGGAAGGTGACGCTAACGCAGATGTAGCGAACGCTCGTCGTATTCTCAAGAAGATTAACATACAGATTCAATCTCGTGGATGGACGTTCAACATTGAGGAAGGCATAACGCTACTACCTGATGTTGACTCCAACCTGATTGTATACAGTGACGACTAATTATCCTTAATGTCTACTTCCGGTCAATACATCTACGTTAACCGAGGTGGCTATGTGTATGACCGAACGAGGCAATAAGACGGCTTTGActcag                                                                                                                   >  1:307094/1‑299 (MQ=255)`  
                                                                                                                                                                                              `gtgAACGAGCTTCTGGGGTGTATCCGTGAACTTCACGTATAAACGATGTAATGGTACGCTCACGCATTTGCTGCGATCTCTCGGCTTATTCTAAACAAGATGAACCGACATATTCCTTCTCGTGGATGGACGTTCAATATTGATGAATGCATAACGCTACTACCTGATGTTTACTCCAACCTGTTTGTATGCAGTTAAGACTATTTATCCCTAATGTCTACTTCCTGTCAATCCATCTACGTTAACCGAGGTGGCTATGTGTATGACCTACCGAGTCAATCAGACCGCTTTGACTCTGGTa                                                                                                                <  1:497709/301‑1 (MQ=255)`  
                                                                                                                                                                                                  `cagACATTCTGGCGGCTATCGGTGAACCTCCGGTATCAACGCTGGAAGGTGACGCTAACGCAGATGTAGCGAACGCTCGGCGTATTCTCAACAAGATTAACCGACAGATTCAATCTCGTGGATGGACGTTAAACATTGAGGAAGGCATAACGCTACTACCTGATGTTTACTCCAACCTGATTGTATACAGTGACGACTATTTATCCATAATGTCTACTTCCGGTCAATCCATCTACGTTAACCGAGGTGGCTATGTGTATGACCGAACGAGTCAATCAGACCGCTTTGACTCTGGTATTAc                                                                                                            <  1:333087/299‑1 (MQ=255)`  
                                                                                                                                                                                                  `atgtgtataagagacagaTCGGTGAACCTCCGGTATCAACGCTGGAAGGTGACGCTAACGCAGATGTAGCGAACGCTCGGCGTATTCTCAACAAGATTAACCGACAGATTCAATCTCGTGGATGGACGTTCAACATTGAGGAAGGCATAACGCTACTACCTGATGTTTACTCCAACCTGATTGTATACAGTGACGACTATTTATCCCTAATGTCTACTTCCGGTCAATCCATCTACGTTAACCGAGGTGGCTATGTGTATGACCGAACGAGTCAATCAGACCGCTTTGACTCTGGTATTAc                                                                                                            <  1:113556/284‑1 (MQ=255)`  
                                                                                                                                                                                                  `aCGACATTCTGGCGTCTATCGGTGAACCTCCTGTATCAACGCTGGAAGGTGACGCTAACCCAGATGTAGCGAACGCTCGGCGTATTCTCAACAAGATTAACCGACAGATTCAATCTCGTGGATGGACGTTCAACATTGAGGAAGGCATAACGCTACTACCTGATGTTTACTCCAACCTGATTGTATACAGTGACGACTATTTATCCCTAATGTCTACTTCCGGTCAATCCATCTACGTTAACCGAGGTGGCTATGTGTATGACCGAACGAGTCAATCAGACCGCTTTGACTCTGGTATTAc                                                                                                            <  1:196256/301‑1 (MQ=255)`  
                                                                                                                                                                                                    `gACATTCTGGCGTCTATCGGTGAACCTCCGGTATCAACGCTGGAAGGTGACGCTAACGCAGATGTAGCGAACGCTCGGCGTATTCTCAACAAGATTAACCGACAGATTCAATCTCGTGGATGGACGTTCAACATTGAGGAAGGCATAACGCTACTACCTGATGTTTACTCCAACCTGATTGTATACAGTGACGACTATTTATCCCTAATGTCTACTTCCGGTCAATCCATCTACGTTAACCGAGGTGGCTATGTGTATGACCGAACGAGTCAATCAGACCGCTTTGACTCTGGTATTACtg                                                                                                          <  1:120810/301‑1 (MQ=255)`  
                                                                                                                                                                                                     `aCATTCTGGGGTCTATCGGTGAACCTACGGTATCAACGCTGGAACGTGACGCTAACGCAGATGTAGCGAACGTTCGGCGTATTCTCAACAAGATTAACCGACAGAGTCAATCTCGTGGATGTACGTTCAACATTTAGGAAGGCATAACGCTACTACCTGATGTTTACTCCAACCTGATTGTATACAGTGACGATTATTTATCCCTAATGTCTACTTCCGGTCAATCCATCTACGTTAACCGAGGTGGCTATGTGTATGACCGAACGAGTCAATCAGACCGCTTTGACTCTGGTATTACtgc                                                                                                         <  1:425705/301‑2 (MQ=255)`  
                                                                                                                                                                                                         `tCTGGCGTCGATCGGTGAACCTCCGGTATCAACGCTGGAAGGTGACGCTAACGCAGATGTAGCGAACGCTCGGCGTATTCTCAACGAGATTAACCGACAGATTCAATCTCGTGGATGGACGTTCAACATTGAGGAAGGCATAACGATACTACCTGATGTTTACTCCAACCTGATTGTATACAGTGACGACTATTTATCCCTAATGTCTACTTCCGGTCAATCCATCTACGTTAACCGAGGTGGCTATGTGTATGACCGAACGAGTCAATCAGACCGCTTTGACTCTGGTATTACTGTGAAc                                                                                                     <  1:458492/301‑1 (MQ=255)`  
                                                                                                                                                                                                                     `cGGTGAACCTCCGGTATCAACGCTGGAAGGTGACGCTAACGCAGATGCAGCGAACGCTCGTCGTATTCTCAACAAGATTAACCGACATATTCAATCTCGTGGATGGACGTTCAACATTGAGGAAGGCATAACGCTACTACCTGATGTTTACTCCAACCTGATTGTATACAGTGACGACTATTTATCCCTAATGTCTACTTCCGGTCAATCCATCTACGTTAACCGAGGTGGCTATGTGTATGACCGAACGAGTCAATCAGACCGCTTTGACTCTGGTATTACTGTGAATATTATTCGtctc                                                                                         <  1:17424/301‑1 (MQ=255)`  
                                                                                                                                                                                                                              `tCCGGTATCAACGCTGGAAGGTGACGCTAACGCAGATGTAGCGAACGCTCGGCGTATTCTCAACAAGATTAACCGACAGATTCAATCTCGTGGATGGACGTTCAACATTGAGGAAGGCATAACGCTACTACCTGATGTTTACTCTAACCTGATTGTATACAGTGACGACTATTTATCCCTAATGTCTACTTCCGGTCAATCCATCTACGTTAACCGAGGTGGCTATGTGTATGACCGAACGAGTCAATCAGACCGCTTTGACTCTGGTATTAcctgtctcttatacacatctccgagccca                                                                                >  1:364811/1‑273 (MQ=255)`  
                                                                                                                                                                                                                                 `gggctcggaaatgggtataagagacagcGCAGATGTAGCGAACGCCCGGCGTATTCTCAAAAAGATAAACCGACAGATTCAATCTCGTGAAT‑GACGTTCAACATTGAGGAAGGCATAACGCTACTACCTGATGTTTACTCCAACCTGATTGTATACAGTGACGACTATTTATCCCTAATGTCTACTTCCGGTCAATCCATCTACGTTAACCGAGGTGGCTATGTGTATGACCGAACGAGTCAATCAGACCGCTTTGACTCTGGTATTACTGTGAACATTATTCGTCTCCGCGACTACGATg                                                                            <  1:377085/274‑1 (MQ=255)`  
                                                                                                                                                                                                                                     `acAACGCTGTAAGGTGACGCTAACGCAGATGTAGCGAACGCTCGGCGTATTCTCAACAAGATTAACCGACAGATTCAATCTCGTGGATGGACGTTCAACATTGAGGAAGGCATAACGCTACTACCTGATGTTTACTCCAACCTGATTGTATACAGTGACGACTATTTATCCCTAATGTCTACTTCCGGTCAATCCATCTACGTTAACCGAGGTGGCTATGTGTATGACCGAACGAGTCAATCAGACCGCTTTGACTCTGGTATTACTGTGAACATTATTCGTCTCCGCGACTACGATgaga                                                                         <  1:319720/300‑1 (MQ=255)`  
                                                                                                                                                                                                                                               `aaGGTGACACCAACGAAAATGTAGCGAACGAGTGGCGTATTCTCAATAAGATGAAACGACAGATTCAATCTCGTGGATGGACGGTCAACATTGAGGAAGGCATAACGTTACTACCTGATGTTTACTCCAACCTTATTGTATACAGTGACGACTATATATCACTAATGTCTACTTCCGGTCAATCCATCGACGTTAACCGAGGTGGCTATGTGTATGACCGAACGAGTCAATCAGACCGCTTTGACTCTGGTATTACTGTGAACATTATTCGTCTCCGCGACTACGATGAGATGCCTGAGTg                                                               <  1:51748/301‑1 (MQ=255)`  
                                                                                                                                                                                                                                                     `aCGCTAACGCAGATGTAGCGAACGCTCGGCGTATCTTCAACAATATTAACCGACAGATTCAATCTCGTGGATGGACGTGTAACATTGATGAAGGCATAACGCTACTACCTGATGTTTACTCCAACCTGATTGTATACAGTGACGACTATTGATCCCTAATGTCTACTTCCGGTCAATCCATCTACGTTAACCGAGGAGGCTATGTGTATGACCGAACGAGTCAATCAGACCGCTTTGACTCTGGTATGACTGTGAACATTATTCGTCTCCGCGACTACGATGAGATGCCTGAGTGCTTCCg                                                         <  1:126182/301‑1 (MQ=255)`  
                                                                                                                                                                                                                                                                          `ctcggagatgtgtataatgaacagttACCCTACAGATTCAATCTCGTGGATGGACGTTCAACATTGAGGACGGCATGACGCTACTACCTGATGTTTACTCCAACCTGATTGTATACAGTGACGACTATTTATCACTAATGTCTACTTCCGGTCAATCCATCTACGTTAACCGAGGTGGCTATGTGTATGACCGAACGAGTCAATCAGACCGCTTTGACTCTGGTATTACTGTGAACATTATTCGTCTCCGCGACTACGATGAGATGCCTGAGTGCTTCCGTTACTGGATTGTCACCAAGGc                                    <  1:449789/277‑1 (MQ=255)`  
                                                                                                                                                                                                                                                                          `aCGCTCGGCGTATTCTCAACAAGATTAACCGACAGATTCAATCTCGTGGATGGACGTTCAACATTGAGGAAGGCATAACGCTACTACCTGATGTTTACTCCAACCTGATTGTATACAGTGACGACTATTTATCCATAATGTCTACTTCTGGTCAATCCATCTATGTTAACCGAGGTGGCTATGTGTATGACCGAACGAGTCAATCAGACCGCTTTGACTCTGGTATTACTGTGAACATTATTCGTCTCCGCGACTACGATGAGATGCCTGAGTGCTTCCGTTACTGGATTGTCACCAAGGc                                    <  1:233514/301‑1 (MQ=255)`  
                                                                                                                                                                                                                                                                                                   `tAACCGTCAGATTCAATCTCGTGGATGGACTTTCAACATTGAGGAAGGCATAACGCTACTACCGGATGTTTACTCCAACCTGATTGTATACAGTGACGACTATTTATCCCTAATGTCTACTTCCGGTCAATCCATCTACGTTAACCGAGGTGGCTATGTGTATGACCGAACGAGTCAATCAGACCGCTTTGACTCTGGTATTACTGTGAACATTATTCGTCTCCGCGACTACGATGAGATGCCTGAGTGCTTCCGTTACTGGATTGTCACCAAGGCTTCCCGTCAGTTCAACAACCGATTc           <  1:141881/301‑1 (MQ=255)`  
                                                                                                                                                                                                                                                                                                   `tAACCGACAGATTCAATCTCGTGGATGGACGTTCAACATTGAGGAAGGCATAACGCTACTACCTGATGTTTACTCCAACCTGATTGTATACAGTGACGACTATTTATCCCTAATGTCTACTTCCGGTCACTCCATCTACATTAACCGAGGTGGCTATGTGTATGACCGAACGAGTCAATAAGACCGCTTTGACTCTGGTATTACTTTGAACATTATTCGTCTCCGCGACTACGATGAGATGCCTGAGTACTTCCGTTACTGTATTATCACCAAGGGTTCCCGTCAGTTCAACAAcctgtct           >  1:295254/1‑296 (MQ=255)`  
                                                                                                                                                                                                                                                                                                   `tAACCGACAGATTCAATCTCGTGGATGGACGTTCAACATTGAGGAAGGCATAACGCTACTACCTGATGTTTACTCCAACCTGATTGTATACAGTGACGACTATTTATCCCTAATGTCTACTTCCGGTCAATCCATCTACGTTAACCGAGGTGGCTATGTGTATGACCGAACGAGTCAATCAGACCGCTTTGACTCTGGTATTACTGTGAACATTATTCGTCTCCGCGACTACGATGAGATGCCTGAGTGCTTCCGTTACTGGATTGTCACCAAGGCTTCCCGTCAGTTCAACAACCGATTc           <  1:359583/301‑1 (MQ=255)`  
                                                                                                                                                                                                                                                                                                    `agcccaCAGATTCAATCTCGTGAATGGACGTTCAACAGTGAGGAAGGCATAACGCTACTACCTGATGTTTACTCCAACCTGATTGTATACAGTGACGACTATTTATCCCTAATGTCTACTTCCGGTCAATCCATCTACGTTAACCGAGGTGGCTATGTGTATGACCGAACGAGTCAATCAGACCGCTTTGACTCTGGTATTACTGTGAACATTATTCGTCTCCGCGACTACGATGAGATGCCTGAGTGCTTCCGTTACTGGATTGTCACCAAGGCTTCCCGTCAGTTCAACAACCGATTCt          <  1:327963/296‑1 (MQ=255)`  
                                                                                                                                                                                                                                                                                                            `gATTCAATCTCTTGGATGGACGTTCAACATTGAGGAAGGCATAACGCTACTACCTGATGTTTACTCCAACCTGATTGTATACAGTGACGACTATTTATCCCTAATGTCTACTTCCGGTCAATCCATCTACGTTAACCGAGGTGGCTCTGTGTATGACCGAACTAGTCAATCAGACCGCTTTGACTCTGGTATTACTGTGAACATTATTCGTCTCCGCGACTACGTTGAGATTCCTGAGTTCTTCCTTTACTTGATTGTCACCACGGCTTCCCGTCAGTTCAACAACCGATTCTATGGGGCa  >  1:492221/1‑301 (MQ=255)`  
                                                                                                                                                                                                                                                                                                            `gATTCAATCTCGTGGATGGACGTTCAACATTGAGGAAGGCATAACGCTACTACCTGATGTTTACTACAACCTGATTGTATACAGTGACGACTATTTATCCCTAATGTCTACTTCCGGTCAATCCATCTACGTTAACCGAGGTGGCTATGTGTATGACCGAACGAGTCAATCAGACCGCTTTGACTCTGGTATTACTGTGAACATTATTCGTCTCCGCGACTACGATGAGATGCCTGAGTGCTTCCGTTACTGGATTGTCACCAAGGCTTCCCGTCAGTTCAACAACCGATTCTTTGGGGCa  >  1:430563/1‑301 (MQ=255)`  

                                                                                                                                                                                                                                                                                                            `|`                                                                                                                                                                                                                                                                                                              
`AGGAGGCCGCACGCACCCGCGCTGCTAACAAAGCCCGAAAGGAAGCTGAGTTGGCTGCTGCCACCGCTGAGCAATAACTAGCATAACCCCTTGGGGCCTCTAAACGGGTCTTGAGGGGTTTTTTGCTGAAAGGAGGAACTATATGCGCTCATACGATATGAACGTTGAGACTGCCGCTGAGTTATCAGCTGTGAACGACATTCTGGCGTCTATCGGTGAACCTCCGGTATCAACGCTGGAAGGTGACGCTAACGCAGATGCAGCGAACGCTCGGCGTATTCTCAACAAGATTAACCGACAGATTCAATCTCGTGGATGGACGTTCAACATTGAGGAAGGCATAACGCTACTACCTGATGTTTACTCCAACCTGATTGTATACAGTGACGACTATTTATCCCTAATGTCTACTTCCGGTCAATCCATCTACGTTAACCGAGGTGGCTATGTGTATGACCGAACGAGTCAATCAGACCGCTTTGACTCTGGTATTACTGTGAACATTATTCGTCTCCGCGACTACGATGAGATGCCTGAGTGCTTCCGTTACTGGATTGTCACCAAGGCTTCCCGTCAGTTCAACAACCGATTCTTTGGGGCA  >  NC_001604.1/24086‑24686`

  

Alignment Legend

Aligned base mismatch/match (shaded by quality score): `ATCG/ATCG` `< 3 ≤` `ATCG/ATCG` `< 9 ≤` `ATCG/ATCG` `< 16 ≤` `ATCG/ATCG` `< 31 ≤` `ATCG/ATCG` `< 38 ≤` `ATCG/ATCG`

Unaligned base: `atcg`    Masked matching base: `atcg`    Alignment gap: `‑`    Deleted base: `‑`
